# Clinical and laboratory diagnosis of monkeypox (mpox): Current status and future directions

**DOI:** 10.1016/j.isci.2023.106759

**Published:** 2023-04-28

**Authors:** Severino Jefferson Ribeiro da Silva, Alain Kohl, Lindomar Pena, Keith Pardee

**Affiliations:** 1 Department of Pharmaceutical Sciences, Leslie Dan Faculty of Pharmacy, University of Toronto, Toronto ON M5S 3M2, Canada; 2 MRC-University of Glasgow Centre for Virus Research, Glasgow G61 1QH, UK; 3 Department of Virology, Aggeu Magalhães Institute (IAM), Oswaldo Cruz Foundation (Fiocruz), 50670-420 Recife, Pernambuco, Brazil; 4 Department of Mechanical and Industrial Engineering, University of Toronto, Toronto ON M5S 3G8, Canada

**Keywords:** Microbiology, Virology

## Abstract

The emergence and rapid spread of the monkeypox virus (MPXV) to non-endemic countries has brought this once obscure pathogen to the forefront of global public health. Given the range of conditions that cause similar skin lesions, and because the clinical manifestation may often be atypical in the current mpox outbreak, it can be challenging to diagnose patients based on clinical signs and symptoms. With this perspective in mind, laboratory-based diagnosis assumes a critical role for the clinical management, along with the implementation of countermeasures. Here, we review the clinical features reported in mpox patients, the available laboratory tests for mpox diagnosis, and discuss the principles, advances, advantages, and drawbacks of each assay. We also highlight the diagnostic platforms with the potential to guide ongoing clinical response, particularly those that increase diagnostic capacity in low- and middle-income countries. With the outlook of this evolving research area, we hope to provide a resource to the community and inspire more research and the development of diagnostic alternatives with applications to this and future public health crises.

## Graphical abstract

**Figure:**
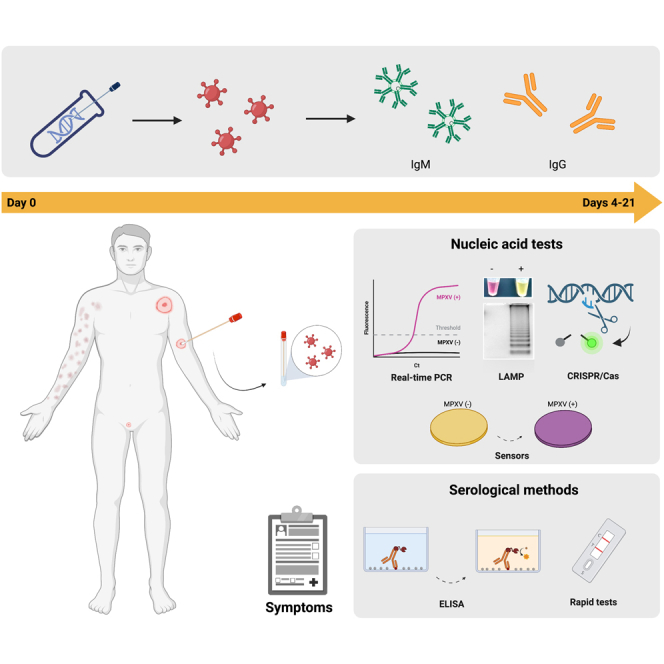

## Introduction

Over the past months, the emergence and rapid spread of monkeypox virus (MPXV) outside of traditionally endemic countries has led to a new viral global threat. The related impact is compounded by the fact that the coronavirus disease 2019 (COVID-19) pandemic is still an ongoing health challenge.^1^^,^^2^ MPXV is a double-stranded DNA virus, a member of the *Orthopoxvirus* genus within the *Poxviridae* family. The virus can be divided into two genetic distinct viral clades: clade I (formerly known as Congo Basin clade) and clade II (former West African clade), which encompasses two phylogenetically distinct subclades, IIa and IIb. The clade I viruses are more virulent, with human case fatality rates during outbreaks in parts of Africa estimated to be around 10%. Clade IIb is responsible for the current global outbreak, although new cases related to clade IIa continue to be reported.^3^ Other *Orthopoxvirus* related species pathogenic to humans include cowpox virus, variola virus, and vaccinia virus.^4^

MPXV was first identified in 1958 in a colony of cynomolgus monkeys (*Macaca fascicularis*) in Copenhagen, Denmark.^5^ Between 1960 and 1968, several outbreaks involving MPXV as an etiological agent were documented in captive monkeys in the Netherlands and the USA.^6^ The first case of MPXV in the human population was reported in 1970 in the Democratic Republic of the Congo in a 9-month-old boy.^7^ Mpox infections remained a disease of the African continent, with sporadic cases diagnosed in forested regions of Central or West Africa and small-scale outbreaks until 2003, when the first cases of infection were reported outside Africa.^8^^,^^9^

In May 2022, a series of mpox cases were reported in Europe, mostly involving men who have sex with men (MSM)^10^^,^^11^^,^^12^ and this emergence has been associated with a steep increase in the number of human mpox infections. When the outbreak of mpox expanded earlier last year, racist and stigmatizing language was observed and reported to World Health Organization (WHO). Following a series of consultations with experts, WHO decided to use a new preferred term named “mpox” as a synonym for monkeypox infection, where both terms will be used simultaneously for one year while “monkeypox” is phased out.^13^^,^^14^ To date, the virus itself remains referred to as MPXV—the International Committee on Taxonomy of Viruses (ICTV) decided to keep the original name to maintain the progress of the scientific literature, at least for now.^15^ As of 11 April, 2023, more than 86,000 cases of mpox infection and 116 deaths have been reported worldwide, most of which involved individuals living in non-endemic countries.^16^ Importantly, it has not been formally demonstrated whether the reported deaths were directly linked with the mpox infection.^16^ However, the rapid spread of the mpox disease led the WHO to declare the current mpox outbreak a Public Health Emergency of International Concern (PHEIC) on July 23, 2022.^17^

Mpox is a zoonotic disease, although its natural animal reservoir(s) remains obscure. Several rodent species from African continent, such as tree squirrels and Gambian pouched rats are currently considered to be strong candidates to act as reservoirs for the virus.^18^^,^^19^ Substantial evidence has been suggested that monkeys and African apes may act as intermediate hosts, and that they can transmit the virus to humans, from which point the disease can spread through close, personal, often skin-to-skin contact between individuals.^20^ Like COVID-19, the present multi-country outbreak of mpox infection demonstrates, yet again, how zoonotic viruses can pose widespread threats to health security, impacting countries beyond their natural endemic range.

Due to the range of conditions that cause skin lesions and because clinical presentation may often be atypical in the current global mpox outbreak, it can be challenging to differentiate the illness on the basis of clinical criteria alone.^21^ Examples of other etiologies with similar-appearing skin lesions include herpes simplex virus, molluscum contagiosum virus, measles virus, enterovirus, varicella zoster virus, and various bacterial skin infections.^21^ In this way, a laboratory-based diagnosis is of paramount importance in assisting physicians in the therapeutic management of patients and for health authorities to deploy countermeasures. Here, we summarize the clinical features and the current laboratory methods used for mpox diagnosis. In addition, we explore novel tools that can provide de-centralized, high-capacity, and low-cost diagnostics for use in remote areas.

## Clinical and epidemiological features

At the clinical and epidemiological level, the features of the classic form of mpox differ of the current pandemic form. In this section, we provide an overview of the clinical and epidemiological characteristics reported in mpox patients. Moreover, we describe key differences between both disease forms. These differences are summarized in Table 1.

**Table 1:** Comparison of the characteristics of the classic form of mpox and the new clinical form

Feature	Classic form (1970s to the present)	Current multicenter outbreak (2022 to the present)
Affected area	Central and West Africa	Countries where mpox is not endemic
Epidemiologic characteristics	Occasional cases and epidemics	Global outbreak under way since May 2022
Dissemination	Mostly intrafamilial and nosocomial	Mostly sexual involving men who have sex with multiple partners
Transmission	Direct contact with an infected animal reservoir, followed by person-to-person transmission	Person-to-person transmission
Clinical presentations	Lesions on the face and extremities, commonly linked with cervical or axillary lymphadenopathy	Perianal lesions, ulcerative lesions, penile and vesicular rash, painful inguinal lymphadenopathy, proctitis, pharyngitis
Clinical evolution	Incubation, prodromal stage, eruption phase with skin lesions	Incubation, prodromal stage (not necessary present), eruption phase with skin lesions, especially on the genitals

Infecting both children and young adults, the classic form of mpox disease can be divided into three different phases. These phases include incubation, prodrome, and the eruptive stage with skin lesions.^22^^,^^23^^,^^24^^,^^25^^,^^26^ The dissemination of the classic form occurs mostly intrafamilial and nosocomial.^27^ In contrast, the current mpox outbreak appears to be mainly transmitted involving MSM populations that have multiple partners.^10^^,^^11^^,^^12^ Clinically, most infections are self-limiting and relatively mild, with symptoms lasting 2–4 weeks. The mean incubation period (from time of exposure to symptom onset) of mpox is currently understood to be about 13 days (range 5–34 days).^28^^,^^29^ Among the infected individuals, the prodromal phase lasts for 1 to 4 days. The most prevalent clinical manifestations described in mpox patients are rash, fever, pruritus, and lymphadenopathy (Table 2).^27^^,^^30^ Lymphadenopathy is a hallmark of mpox infection and essentially is used to distinguish it from other poxviruses, including smallpox or chickenpox.^27^ Typically, lymphadenopathy occurs in submandibular glands, axilla, groin, and neck.^31^ Other manifestations include fatigue, sore throat, headache, cough, myalgia, photophobia, arthralgia, difficult breathing, conjunctivitis, nausea/vomiting, and diarrhea.^30^^,^^32^^,^^33^ The eruptive phase, which usually occurs around the lasts 14 to 28 days, is characterized with the development of skin lesions with a centrifugal distribution, mostly concentrated on the face and distal extremities.^27^ The evolution of lesions progress through four stages, that include macules, papules, vesicles, and pustules.^27^

**Table 2:** Clinical characteristics of mpox patients

Reference	Pittman et al.^34^	Yinka-Ogunleye et al.^35^	Huhn et al.^25^	Adler et al.^36^	Patel et al.^30^	Thornhill et al.^37^
Country	Democratic Republic of the Congo	Nigeria	USA	United Kingdom	United Kingdom	International collaborative group (43 sites in 16 countries)
Medical description	216 patients	122 patients	37 patients	7 patients	197 patients	528 patients
Fever	18.5%	79%	87%	42%	61.9%	62%
Rash	99.5%	88%	97%	100%	13.7%	95%
Headache	23.6%	79%	65%	–	–	27%
Myalgia	6.9%	58%	56%	–	31.5%	31%
Malaise	85.2%	50%	–	–	–	–
Sore throat	78.2%	58%	60%	–	16.8%	–
Chill	44.9%	65%	71%	–	–	–
Adenopathy	57.4%	69%	71%	71%	57.9%	56%

Lesion stages have appeared simultaneously and progressed sequentially during the clinical course of the disease.^30^ Specifically, these lesions have commonly been found in areas of the body like the face, mucous membranes, palms, and soles.^27^ In the current mpox outbreak, the lesions appear in an unusual distribution, especially on the genitals.^27^ A growing body of data have demonstrated that the symptom severity and disease duration are proportional to the density of skin lesions.^27^ In humans, severe complications of infection include encephalitis, pneumonia, secondary skin infection, and ocular disease leading to loss of vision.^33^^,^^38^ Populations at high risk to develop severe disease and present more severe complications include: neonates, children, pregnant women, and immunocompromised persons, especially individuals infected with human immunodeficiency virus (HIV).^27^^,^^39^^,^^40^ Among the infected patients, approximately 35% require clinical care.^33^ Historically, the case fatality ratio of the classic form of mpox ranges from 1% to 15%,^27^ while, in the recent multi-country outbreak, the overall case fatality rate appears to be lower (0%–4%) in the human population.^27^^,^^33^

## Laboratory-based diagnosis of mpox

**Figure 1:**
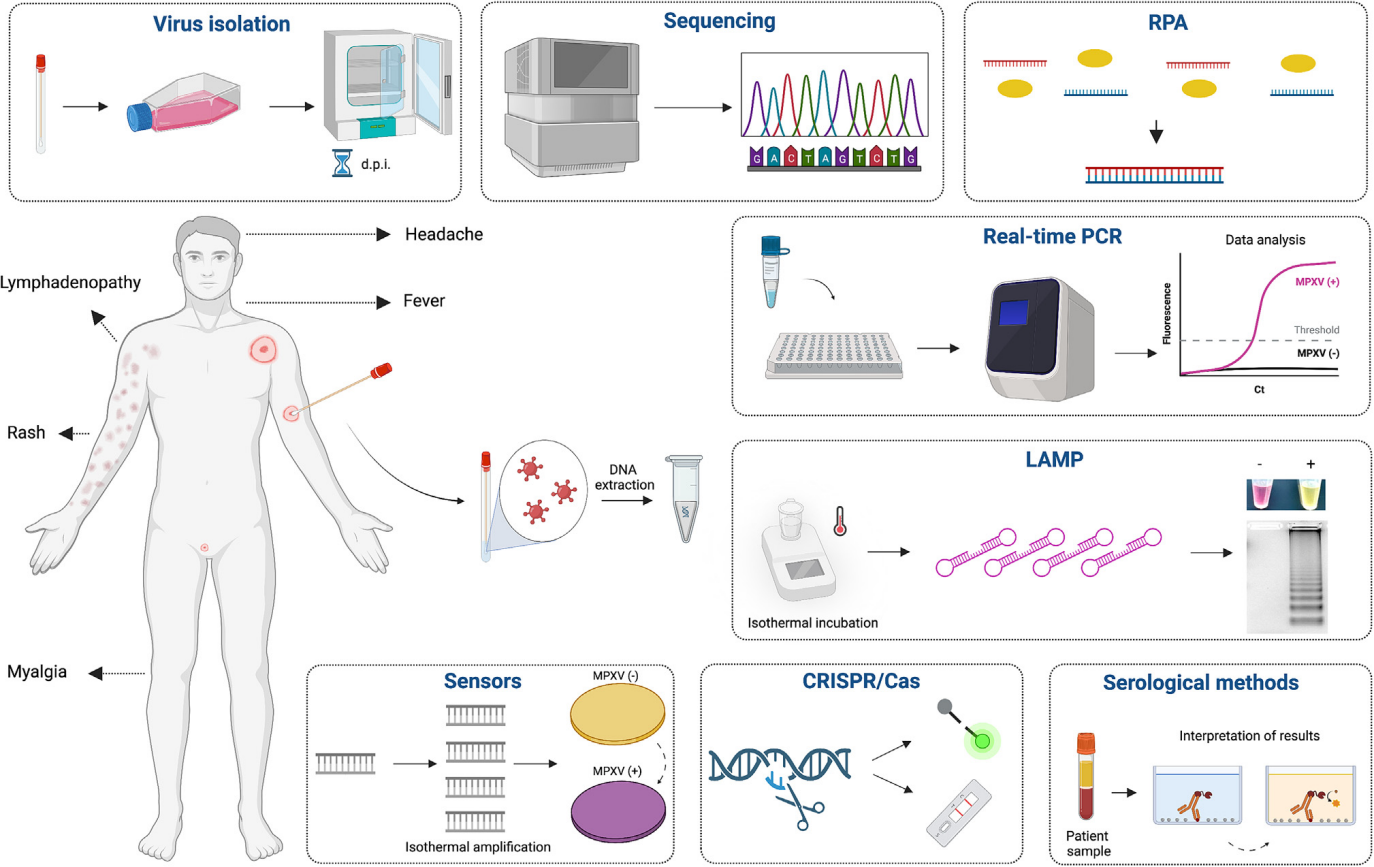
Clinical symptoms commonly reported in patients and currently strategies for mpox diagnosis Among mpox infected patients, the most prevalent clinical manifestations are rash, headache, myalgia, fever, and lymphadenopathy (left figure), with lesion density correlated with disease severity. To better understand the virus, virions can be isolated from patient samples for characterization. Diagnosis of mpox can be done in many ways; here we highlight lab-based nucleic acid testing, including emerging isothermal methods that have the potential to be brought to the point of need.

Laboratory virology methods are critical for a correct diagnosis and to investigate the population level prevalence of infection. Results from these tests guide physicians and health authorities in the management, control, and prevention of mpox cases as an outbreak evolves and spreads. To date, the unequivocal confirmation of mpox infection is done through the use of direct and indirect diagnostic methods (Figure 1). In direct tests, the clinical specimen is investigated for the presence of the virus, viral nucleic acids, or antigens. For this purpose, nucleic acid amplification tests (NAATs) are most commonly applied to identify the DNA (deoxyribonucleic acid) sequences that comprise the genetic material of the virus. In contrast, indirect MPXV tests detect the patient immune response against the viral infection. In the section below, we provide the basic information related to biosafety, sample collection, transport, and storage of biological materials containing MPXV. Moreover, we summarize and explore the different detection strategies being developed or used for mpox diagnosis, discussing their advances, principles, advantages, and limitations. We also highlight the methods with potential for future applications that may serve ongoing needs.

### Biosafety considerations for diagnostic testing

According to the U.S. Centers for Disease Control and Prevention (CDC), laboratories that process and perform mpox diagnosis using patient samples, such as swabs of lesion surface and exudate, and lesion crusts, should have the appropriate infrastructure and level of biosafety, and be performed only by trained professionals.^41^ Non-propagative diagnostic tests, such as NAATs and some serological assays can be performed in biosafety level 2 (BSL-2) laboratories provided that the initial processing of specimens takes place in a certified biological safety cabinet, especially if there is potential to generate aerosols.^41^ However, procedures that involve propagative virus work, such as virus culture or isolation, should be done only in laboratories equivalent to BSL-3 using validated safety practices and following biocontainment precautions.^41^ Importantly, biosafety regulations for national laboratories working with MPXV specimens must be conducted in accordance with a designated regulatory agency (e.g., U.S. CDC in the USA; European Center for Disease Prevention and Control [ECDC] in European countries) and, therefore, may vary for each country. For example, MPXV is not considered a bioterrorism agent according to the U.S. list of bioterrorism agents,^42^ but it is considered an “agent with high threat for deliberate release” according to the European Union task force on Bioterrorism (BICHAT).^43^

Since the emergence of MPXV, nosocomial infection of healthcare workers has been documented in different parts of the world.^44^^,^^45^ Within this perspective, measures should be taken to minimize the risk of laboratory transmission based on the risk assessment when testing routine clinical specimens from suspected or confirmed mpox patients.

### Specimen collection, transport, and storage

Choosing the correct specimens for diagnosis tests is a critical step in a reliable and accurate diagnosis. According to the WHO criteria, the recommended sample type for laboratory investigation of mpox infection is skin lesion material, including roofs from more than one lesion roofs (e.g., lesion crusts) and swabs of lesion surface and/or exudate.^21^ Two lesions of the same morphology should be collected in one single tube, preferably from different areas.^21^ Importantly, lesions, crusts, and vesicular fluids should not be mixed in the same tube. If resources allow, two tubes may be collected to minimize risk of poor DNA concentration or presence of inhibitors. In addition to lesion samples, the collection of an oropharyngeal swab is also encouraged. Importantly, data on the accuracy of this type of sample for mpox diagnosis is scarce, and therefore a negative throat swab sample should be interpreted with caution.^21^

Collection of alternative sample types for research purposes can also be considered.^21^ These samples may include rectal and/or genital swab, semen, and urine on indication based on clinical presentation.^21^ Ethylenediaminetetraacetic acid (EDTA)-treated blood may support detection of MPXV, but this type of sample may not contain the high level of virus found in lesion samples, since any viremia occurs early in the clinical course of infection, usually in the prodromal phase, and before the appearance of skin lesions.^21^ Sample collection should be performed by health professionals following adequate standard operating procedures (SOPs) and with appropriate donning and doffing of personal protective equipments (PPEs).^21^ Notably, these additional sample types are indicated for only routine diagnostic purposes and do not need to be collected outside of research settings.^21^

Samples collected for mpox investigation should be refrigerated (2–8°C) or frozen (−20°C or lower) within an hour after collection and transported to the diagnostic laboratory as soon as possible.^21^ Correct handling and storage of specimens during transportation is a critical step for accurate diagnostic testing. If transport exceeds seven days for the sample to be processed, all samples should be stored at −20°C or lower. Longer term sample storage (> 60 days from collection) is recommended at −70°C.^21^ This storage considerations are important to prevent false-negative results. Several factors, such as poor quality of specimen, improper handling or shipping, or technical reasons inherent to the assay (e.g., DNA extraction failure), can affect the diagnostic performance and quality control in a reference laboratory.

### Clinical laboratory findings and biomarkers

Besides the laboratory techniques for MPXV diagnostics discussed throughout this review, previous studies have investigated biochemical and blood chemical alterations involved during the clinical progression in mpox patients. The levels of these biomarkers fluctuate depending on the clinical stage of the disease. Compared to healthy persons, clinical biomarkers associated with mpox infection include thrombocytopenia, leukocytosis, hypoalbuminemia, low blood urea nitrogen level, and elevation of transaminase level. Among these biomarkers, thrombocytopenia was determined as the most common biochemical alteration in patients.^25^^,^^34^^,^^35^ While previously suggested as biomarkers for MPXV infection, more recent work has established that elevated aspartate aminotransferase (AST) and alanine aminotransferase (ALT) expression have poor prognostic value.^34^ Increased levels of several cytokines have been reported in mpox patients (regardless of disease severity), including interleukin (IL)-1RA, IL-1β, IL-2R, IL-4, IL-5, IL-6, IL-8, IL-13, IL-15, IL-17, CCL5, and CCL2.^46^ In severe cases (defined as having > 250 lesions), concentrations of IL-10, IL-2R, CCL5, and granulocyte-macrophage colony-stimulating factor (GM-CSF) were higher than those in patients with less severe disease.^46^ Importantly, no single biomarker can be used to confirm or discard a case of mpox infection, thus a laboratory diagnostic test should be conducted for all suspected cases.

### Virus isolation

In 1958, MPXV was first isolated from pustules observed in a colony of cynomolgus monkeys.^5^ Here, monkey kidney (HeLa) and human amnion cells were used for virus isolation. In humans, MPXV was first isolated on a patient with smallpox-like disease from skin lesions in 1970.^7^ The isolation was conducted by infecting immortalized cell lineages including PEK (pig embryonic kidney cells), HEP-2 (*Homo sapiens* epithelial carcinoma cells), and Vero (African green monkey kidney). Briefly, the cellular infectivity of the isolated virus was confirmed by the presence of the cytopathic effect (CPE).^7^ Culture-based methods for MPXV detection have been used in public health and research laboratories in different countries around the world, but virus isolation is not officially recommended by the WHO as a routine diagnostic technique because it has several drawbacks, which include being time-consuming, providing low sensitivity, and requiring BSL-3 infrastructure.^21^ MPXV is also grown in several cell lines, such as Vero, Vero E6, Vero 76, BSC-1, HEP-2, PEK, MA-104, HeLa, BSC-40, LLC-MK2, and Balb/3T3 clone A31.^47^ Typically, these cells lineages are susceptible, and therefore, have potential for use in scientific studies in order to evaluate potential therapeutic agents and study basic aspects of MPXV biology.

Since the beginning of the current multi-country outbreak, several studies have demonstrated the isolation of MPXV from different types of specimens. In one of the earliest reports, Lapa and colleagues documented the MPXV isolation from a semen specimen collected in the early phase of infection from a patient with prolonged seminal viral shedding.^48^Here, for the virus isolation, the authors inoculated semen collected on day 6 after symptom onset in Vero E6 cells.^48^ Clear cytopathic effect was visualized 48 h after the inoculation and MPXV replication was confirmed by real-time PCR.^48^ Similarly, a recent report described the isolation of viable MPXV from anal and urethral swabs using Vero E6 cells.^49^

### Electron microscopy

MPXV particles exhibit a brick-shaped (200–250 nm) or ovoid format and have a complex internal structure, including a double-stranded DNA (dsDNA) genome (∼197 kilobases) and associated enzymes.^50^^,^^51^^,^^52^ Given the distinctive morphology of the virus, electron microscopy (EM) has been applied to observe and identify virus particles after isolation in culture-based systems. In several studies, EM has been used to evaluate specimens for all progeny virions at various stages of assembly (e.g., immature and mature MPXV particles) in the cytoplasm of infected cells.^53^^,^^54^ Despite the value for research studies, EM is impractical as a routine diagnostic technique to detect MPXV in infected patients.

### Genome sequencing

In addition to the conventional diagnosis of mpox infection, whole genome DNA sequencing has been used for tracking changes in the viral genome over time and tracing transmission patterns during the current epidemiological scenario. However, given the inherent limitations of genome sequencing, such as the high cost of reagents and infrastructure, and the need for specialized training, the technique unsuitable for clinical practice. Currently, only a small percentage of patient samples are being selected for DNA sequencing. Here, sequencing protocols based on metagenomic approach and next-generation sequencing (e.g., Illumina and MinION) tools are being applied to generate MPXV genome sequences from clinical samples.^55^^,^^56^^,^^57^^,^^58^ Similar to challenges that were faced during the COVID-19 pandemic, here, genomic surveillance of circulating lineages has been critical to guide health authorities and governments in decision making with respect to the implementation of public health measures to reduce the transmission.^59^^,^^60^^,^^61^^,^^62^ Accordingly, constant genomic surveillance should be implemented on a large scale in order to track genetic changes, establish policies, and inform countermeasure development to break the chain of MPXV transmission.

### Real-time PCR

**Figure 2:**
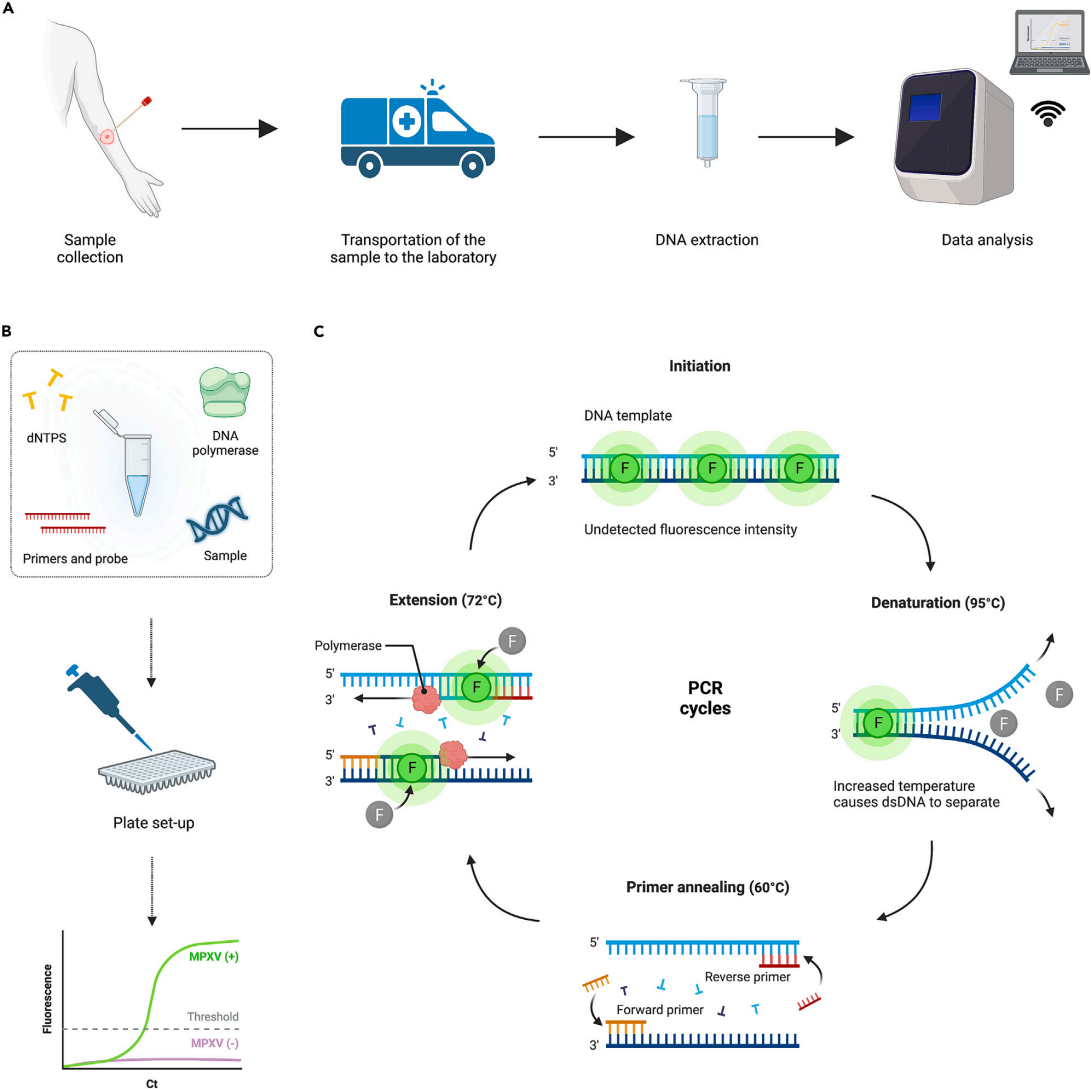
Real-time PCR workflow for MPXV detection (A–C) Common steps required for the diagnosis of MPXV with real-time PCR (a). Real-time PCR assay and plate set-up (b). Principle of real-time PCR: The double-stranded DNA (dsDNA) is used as a template. The initial denaturation step is carried out at the beginning of PCR to separate the double-stranded template DNA into single strands so that the primers can bind to the target region. At the annealing stage, the reverse primer binds to the sense strand of dsDNA in a sequence-specific strategy, and the forward primer and a dually labeled probe bind to the antisense strand of the DNA. During the extension phase, the DNA polymerase extends the forward primer and, in the process, hydrolyzes the probe, resulting in the release of the fluorophore. Then, following excitation, fluorescence emission can be captured by the real-time instrument and data visualization can be done using designed software’s. After ∼ 40 cycles of amplification, the reaction is complete (c).

According to WHO and U.S. CDC guidelines, any individual meeting the definition for a suspected case should be offered testing.^21^^,^^41^ Currently, real-time PCR is the gold standard molecular method for lab-based diagnosis of mpox, for samples from either patients or wild animals (Figure 2).^21^^,^^63^ Since the emergence of MPXV, several real-time PCR assays have been developed for the diagnosis of this virus.^64^ These real-time PCR assays have been designed for different targets in the MPXV genome (G2R, B7R, F3L, B6R, N3R, and TNF receptor gene), and the diagnostic validation has been evaluated using clinical samples (PCR protocols and primer sequences are summarized in Table 3). With regard to the limit of detection (LoD), the majority of real-time PCR tests provide LoDs ranging from 250 to 10 copies per reaction.^64^^,^^65^^,^^66^ To date, there are seven diagnostic tests based on real-time PCR that have been granted Emergency Use Authorization (EUA) by the U.S. Food and Drug Administration (FDA) (https://www.fda.gov/medical-devices/emergency-use-authorizations-medical-devices/monkeypox-emergency-use-authorizations-medical-devices).

**Table 3:** Real-time PCR assays for MPXV diagnostics

Target	Sequence (5’ – 3′)	Limit of detection (LoD)	Validation with real-life samples	Reference
TNF receptor gene^a^	Forward: GGAAAATGTAAAGACAACGAATACAG Reverse: GCTATCACATAATCTGGAAGCGTA Probe: FAM-AAGCCGTAATCTA<BHQ-1dT>GTTGT CTATCGTGTCC-Spacer C6-3′	–	Yes	^64^
G2R	Forward: CACACCGTCTCTTCCACAGA Reverse: GATACAGGTTAATTTCCACATCG Probe: FAM-AACCCGTCGTAACCAGCAATACATTT-BHQ1	∼8.2 genome copies	Yes	Li et al.^65^
B7R	Forward: ACGTGTTAAACAATGGGTGATG Reverse: AACATTTCCATGAATCGTAGTCC Probe: TAMRA-TGAATGAATGCGATACTGTATGTGTGGG-BHQ2	50 copies	Yes	Shchelkunov et al.^66^
F3L	Forward: CTCATTGATTTTTCGCGGGATA Reverse: GACGATACTCCTCCTCGTTGGT Probe: 6FAM-CATCAGAATCTGTAGGCCGT-MGBNFQ	50–250 copies	Yes	Kulesh et al.^63^
G2R	Forward: TGTCTACCTGGATACAGAAAGCAA Reverse: GGCATCTCCGTTTAATACATTGAT Probe: FAM-CCCATATATGCTAAATGTACCGGTACCGGA-BHQ1	∼40.4 copies	Yes	Li et al.^65^
B6R	Forward: ATTGGTCATTATTTTTGTCACAGGAACA Reverse: AATGGCGTTGACAATTATGGGTG Probe: MGB/DarkQuencherAGAGATTAGAAATA-FAM	∼10 viral copies	Yes	Li et al.^67^
N3R	Forward: AACAACCGT CCTACAATTAAA CAACA Reverse: CGCTATCGAACCATTTTTGTAGTCT Probe: 6FAM-TATAACGGCGAAGAATATACT-MGBNFQ	50–250 copies	Using rodent samples	Kulesh et al.^63^

FAM: fluorescein.

a U.S. CDC recommended protocol for testing patient samples.

With the clinical presentation caused by mpox infection similar to those of other infectious agents, differential diagnosis is a critical step and, with this in mind, multiplex real-time PCR methods provide a molecular strategy to simultaneously detect and distinguish different infectious agents, orthopoxviruses, and MPXV clades as well (e.g. Western Africa and Congo Basin).^68^^,^^69^^,^^70^ In response to the recent mpox outbreak, Huo and colleagues developed two multiplex real-time PCR assays with high sensitivity and specificity for simultaneous detection and differentiation of MPXV IIa, IIb, and I clades and the B.1 lineage.^70^ Another new PCR-based strategy, named the *pan-Orthopoxvirus* assay, was previously designed based on a broad-range PCR coupled with electrospray ionization mass spectrometry (PCR/ESI-MS) to detect MPXV from spiked human and animal specimens.^68^ Taken together, these multiplex formats have a number of advantages to the laboratory routine in comparison to the monoplex format, especially for use in well-resourced areas with circulation of other orthopoxviruses.

### Loop-mediated isothermal amplification (LAMP)

Despite RT-qPCR being the current gold standard technique for the diagnosis of mpox infection, it has several drawbacks, including long sample processing time, requires technical expertise, reliable access to electricity, and utilizes a sophisticated thermocycler for detection and amplification of the viral genome.^71^^,^^72^^,^^73^ These limitations make the method unsuitable for distributed applications, particularly in low- and middle-income areas.^71^ Point-of-care (POC), reliable, easy-to-use assays will be absolutely critical for combating mpox, especially as the disease moves through low- and middle-income countries. With this in mind, isothermal methods such as loop-mediated isothermal amplification (LAMP) are perhaps among the most promising techniques for rapid detection of MPXV.^74^^,^^75^ LAMP is a rapid, low-cost, simple, and powerful method for the rapid amplification of nucleic acid at a single and isothermal temperature (e.g. 60°C–65°C),^74^^,^^76^^,^^77^ which means that assays can be performed without expensive equipment. Together, these characteristics are highly desirable for POC diagnostic applications in regions with limited laboratory infrastructure.

**Figure 3:**
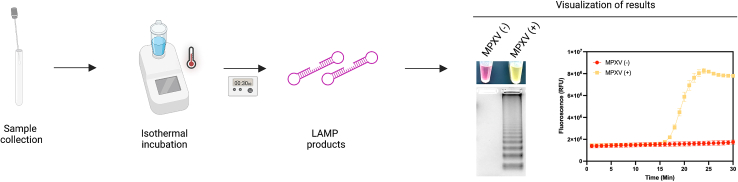
Amplification of nucleic acids using the LAMP technique

The LAMP method was first described by Notomi et al.^76^ and has since undergone many improvements and adaptations to provide robust detection of pathogens for applications in animal, plant, and human health.^76^ LAMP is an isothermal nucleic acid amplification technique that often employs a set of four or six different primers, which specifically bind to complementary sequences in the genome.^78^ Following isothermal incubation for as little as 20 min, results can be easily interpreted by the naked-eye through analysis of color change using dsDNA binding dyes (e.g., SYBR green, calcein, hydroxynaphthol blue dye [HNB], etc).^71^ Results can be also monitored through a variety of other readouts methods, including turbidity measurement, real-time fluorescence, pH indicator (e.g., phenol red, cresol red, and neutral red), gel electrophoresis, and other approaches (Figure 3).^74^ Efforts to decrease the cost and simplify the LAMP workflow are in progress using in-house-produced enzymes. For example, *Bst* DNA polymerase large fragment, a main component of the LAMP reaction, has been expressed and purified from *Escherichia coli* BL21(DE3)^79^^,^^80^ and cell-free (CF)-based expression systems.^81^ Notably, homemade production of enzymes solves important practical limitations in the deployment of molecular diagnostics to the field and demonstrates how distributed manufacturing can increase the diagnostic capacity of low- and middle-income countries.

Considering its features of high specificity and sensitivity, simple operation, and fast amplification, LAMP assays have been developed for the diagnosis of many infectious diseases.^82^^,^^83^^,^^84^^,^^85^^,^^86^^,^^87^ This includes LAMP assays for other poxviruses, including sheeppox virus (SPPV), goatpox virus (GTPV), and lumpy skin -disease virus (LSDV).^88^^,^^89^^,^^90^ In response to the recent multi-country mpox outbreak, a global effort has been mounted to develop LAMP platforms for MPXV detection.^91^^,^^92^^,^^93^ These in-house LAMP protocols and primer sequences are summarized in Table 4.

**Table 4:** LAMP assays for MPXV diagnostics

Target	Sequence (5’ – 3′)	Limit of detection (LoD)	Validation with real-life samples	Reference
D14L	F3-C: TGGGTGGATTGGACCATT B3-C: ATGGTATGGAATCCTGAGG FIP-C: TGGGAGCATTGTAACTTAT AGTTGCCCTCCTGAACACATGACA BIP-C: ATCCTCGTATCCGTTATGTCTTCC CACCTATTTGCGAATCTGTT LOOP-F-C: GATATTCGTTGATTGGTAA CTCTGG LOOP-C-C: GTTGGATATAGATGGAG GTGATTGG	10^2.4^ copies	Yes	Iizuka et al.^91^
ATI	F3-W: TACAGTTGAACGACTGCG B3-W: AGTTCAGTTTTATATGCCGAAT FIP-W: CCGTTACCGTTTTTACAA TCGTTAATCAATGCTGATATGG AAAAGAGA BIP-W: ATAGGCTAAAGACTAGAATCA GGGATTCTGATTCATCCTTTGAGAAG LOOP-F-W: GATGTCTATC AAGATCCATGATTCT LOOP-C-W: TCTTGAACGATCGCTAGAGA	10^3^ copies	Yes	Iizuka et al.^91^
A27L	A27L-1F3: TTCTTGTATTTGTGGGAACAT A27L-1B3: GATGGATGAGGAAGTGCC A27L-1FIP: CCATCCCCCACCTAATAA TGATAAATAGGATCTTCTAAT GGATTGTATGG A27L-1BIP: AATTGGTTGGTCCTC CTTATCTCCACAAGCATTTGTCTAAGCCTA A27L-1LB: TCCAGTAGCATGTGGTTC	20 copies	Using simulated clinical samples	Feng et al.^92^
F3L	F3L-1F3: TCTCGTTTAGATTTTCCATCTG F3L-1B3: TCTTTTGATGATGTTATTCCGG F3L-1FIP: TGGGGCCTAGTAACTCTC CTACCCTTATCGAATACTCTTCCGT F3L-1BIP: TCAATACGAAAAGACC AATCTCTCCAAAGGTGTTAACCCTGTCAC F3L-1LF: ATTTTATGCCTGTGTAGACATTG	20 copies	Using simulated clinical samples	Feng et al.^92^
N4R	F3: GCGAATAAGACAGTGCGATT B3: TCATACAGAACATCTACAGGAT FIP: GACCAAAGATCGAGGTCGT CGATGGAGTCGGTAGATTTCATG BIP: TGGATTAGGTGTTGAC TGTTATGTTCACAAATTGGTTCAAGGAGAA LF: GAAACTGCTCATCGACAGC LB: CTAGAACCAGTTGTTGACAGGA	2 × 10° copies/μL	Yes	Yu et al.^93^

### Recombinase-based isothermal amplification assays

Recombinase polymerase amplification (RPA), developed by TwistDx (Cambridge, UK), and recombinase-aid amplification (RAA) by ZC Bioscience (Hangzhou, China) are isothermal amplification methods, in which an enzymatic-based DNA amplification can be achieved at constant temperature (optimally around 37 to 42°C) in just a few minutes (3–15 min).^94^ In both methods, the amplification process is initiated by a primer recombinase-complex. This complex then invades the double strand DNA (dsDNA) at target sequences homologous to the primer, enabling the sequence-specific recognition of the template target sites by oligonucleotide primers. This step is followed by strand-displacing DNA synthesis, resulting in the exponential amplification of the target.^94^ In the case of real-time detection, the probe is added to the reaction system and its cleavage can result in a fluorescent signal.^94^ Other detection methods include gel electrophoresis or lateral flow assay of the reaction product.^95^ Considering the advantages of rapid amplification, simple operation, high sensitivity, and compatibility with multiplexing, recombinase-based methods (RPA/RAA) have the potential to create field-applicable diagnostics for use in resource-limited settings. Not surprisingly, studies have already reported the development of RPA/RAA methods for MPXV detection.^96^^,^^97^

### Sensors

Sensors are tools that respond to a stimulus, such as chemical, physical, or biological, and generate a signal that can be measured or interpreted through an output method (e.g. colorimetric, fluorescence, electrochemical, etc).^98^ Given the simple operation, low cost, versatility, rapid amplification, and capacity for high throughput testing, low-burden gene circuit-based sensors have the potential to eliminate the bottlenecks faced by real-time PCR, especially for use in remote areas with limited laboratory infrastructure.^99^^,^^100^^,^^101^ Among the various types of biosensors, in recent efforts have focused on moving clinical-grade sensors into the field for use in clinical practice. In terms of application, most sensors currently under development for MPXV diagnostics are based on methods previously validated for the diagnosis of other pathogens, such as Zika virus,^74^^,^^99^ SARS-CoV-2,^102^ and Ebola virus.^101^

**Figure 4:**
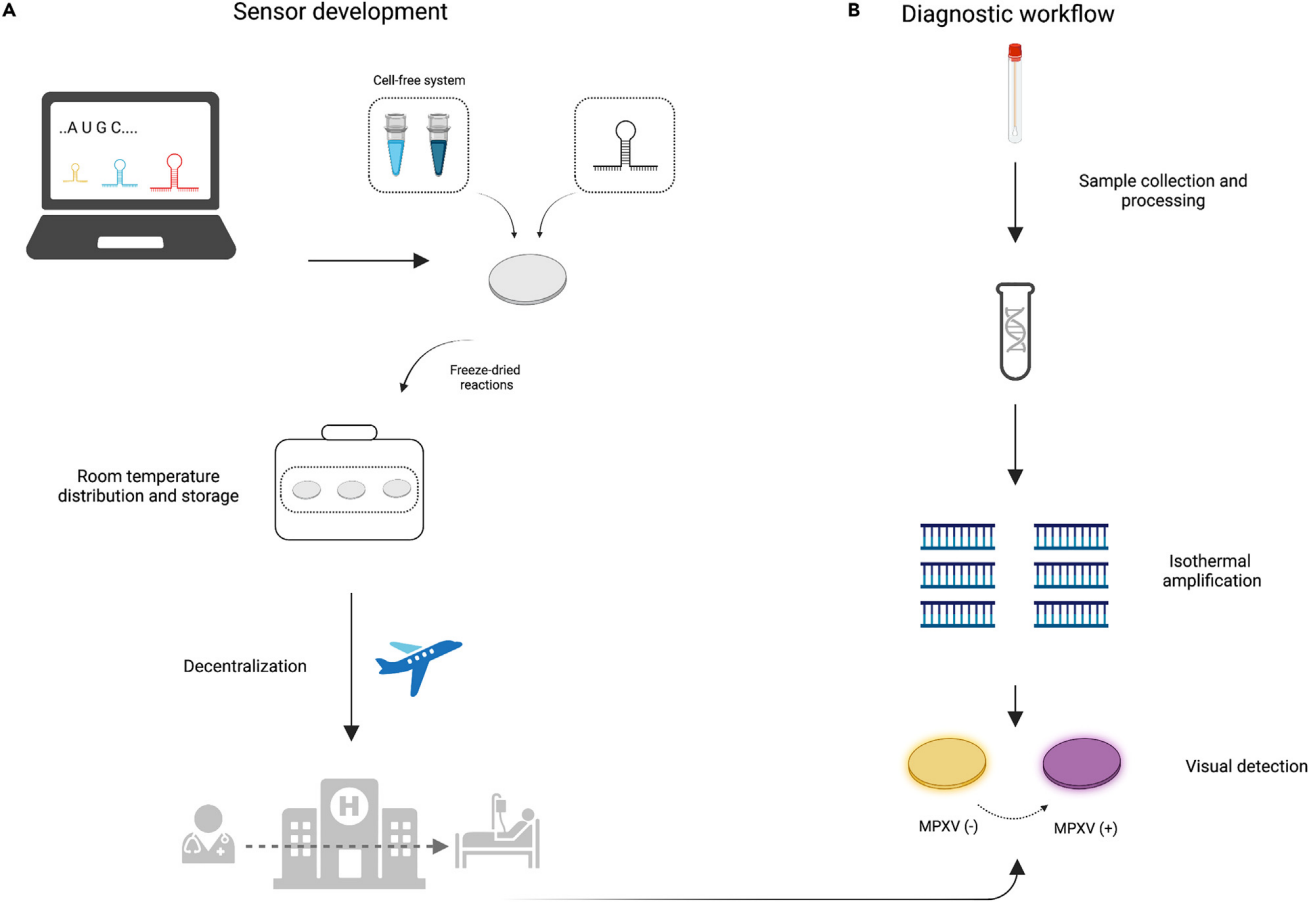
Workflow for the rapid design and testing of paper-based sensors Using sequence information from online databases, toehold switch-based sensors are designed in silico using specific algorithms. Once synthesized, the resulting sequence-specific toehold sensors can be assembled and embedded into paper and freeze-dried along with a cell-free system (e.g., transcription and translation components) to be deployed in the field settings as a stable platform (A). For the diagnostic workflow, extracted DNA from patient samples is amplified using isothermal techniques (e.g., LAMP [loop mediated isothermal amplification], RPA [recombinase polymerase amplification], and NASBA [nucleic acid sequence based amplification]). Following the isothermal amplification, the detection of the appropriate target is indicated by a color change in the paper disc from yellow to purple (B).

In the past few years, and as part of collaborative consortia, we have contributed to the development of new, toehold switch sensor-based diagnostics in response to the series of pathogen outbreaks that have recently affected global public health. Our effort has included the development and validation of point-of-care (POC) tests in response to the Ebola outbreak in Africa,^101^ the Zika and chikungunya epidemics in the America,^99^^,^^100^^,^^103^ and the COVID-19 pandemic,^102^ which provided proof-of-concept work for the use of cell-free protein expression (CF) reactions for the diagnosis of emerging and re-emerging pathogens. Briefly, our sensor platform works as programmable RNA sensors (toehold switches) that activate the translation of a reporter gene (e.g., β-galactosidase [LacZ] or green fluorescent protein [GFP]) in presence of a RNA trigger sequence.^99^^,^^101^ Specifically, toehold switches contain a hairpin structure that blocks downstream translation by sequestering the start codon and the ribosomal binding site (RBS).^99^^,^^101^ If the target sequence is present in the sample, it activates the translation of a reporter protein to create an optical signal that mediates a color change in the reaction (Figure 4).^99^ In addition molecular diagnostics, previous work has showed that CF systems can be used to detect various other analytes, such as water contaminants,^104^ antibiotics (e.g., tetracycline^105^), toxic metals (e.g., mercury^106^), biomarkers (e.g., hippuric acid^107^), and endocrine disruptors in human blood and urine.^108^ Taken together, these efforts have demonstrated the potential of low-burden sensors in global crises, POC diagnostics, and use in clinical laboratories. As of the date this review was written, no such sensing platforms had been reported for MPXV diagnostics; however, we anticipate that new sensing technologies will be developed.

#### CRISPR/cas-based systems

CRISPR-based sensing is another emerging category of signal detection methods for nucleic acids that provide high specificity and sensitivity, simple device structure, and excellent compatibility with multiple readouts methods including lateral flow assays (LFAs) or fluorescence.^109^^,^^110^^,^^111^^,^^112^^,^^113^ Over the past few years, substantial progress has been in the design of molecular diagnostics using CRISPR/Cas components from the microbial adaptive immune system.^109^ Briefly, in their natural context, the CRISPR/Cas system recognizes viral nucleic acids on the basis of their sequence (DNA or RNA) and subsequently eliminate them using endonuclease activity associated with the Cas enzyme.^109^ Among the diverse CRISPR systems, two categories were rapidly used for diagnostic proposals, which include SHERLOCK (**s**pecific **h**igh-sensitivity **e**nzymatic **r**eporter un**lock**ing, Cas13a)^111^ and DETECTR (for **D**NA **e**ndonuclease-**t**argeted **C**RISPR *trans* **r**eporter, Cas12a).^114^

**Figure 5:**
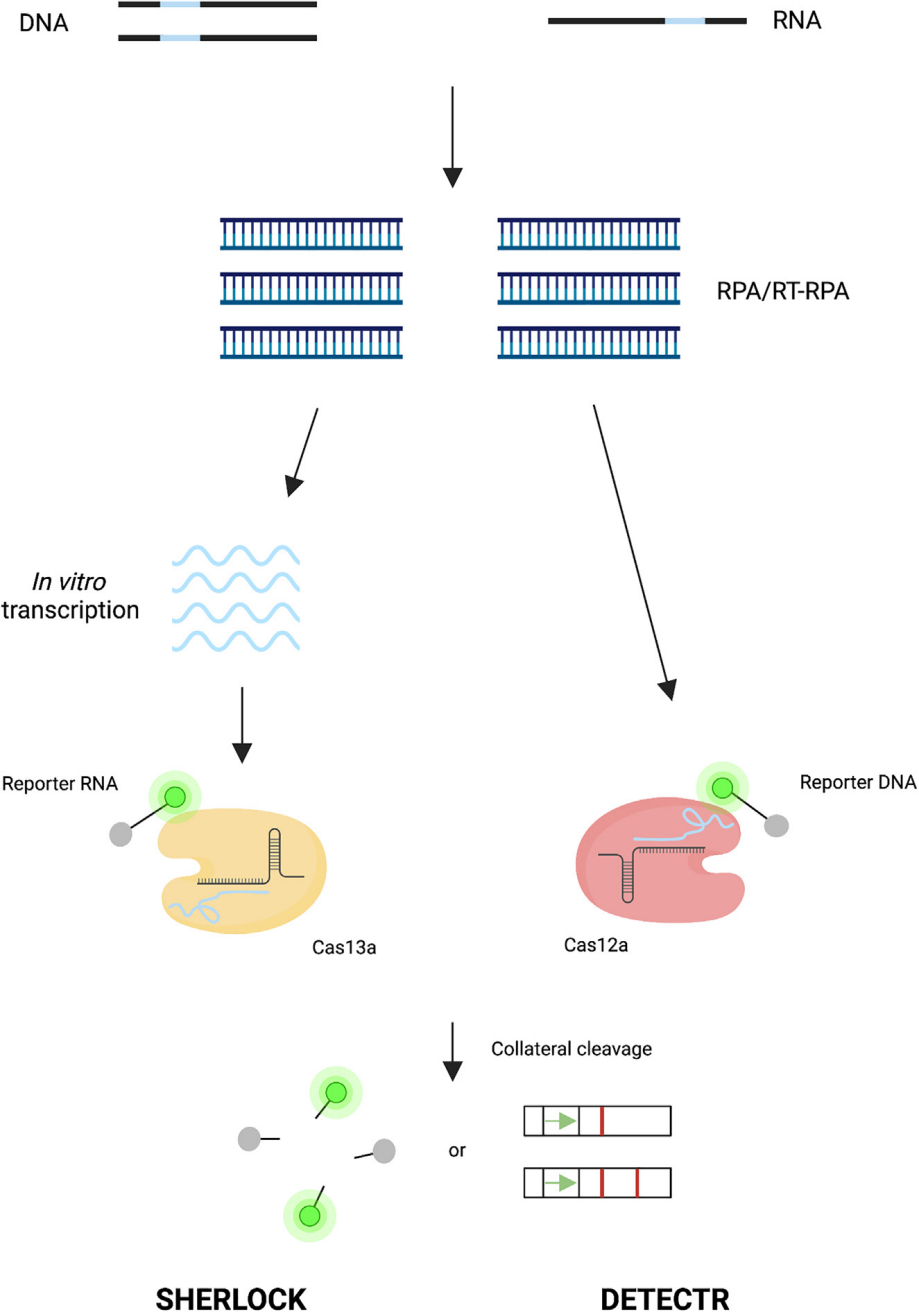
Principle of CRISPR-Cas technology for acid nucleic (DNA and RNA) detection Overall, CRISPR-Cas–based diagnostics combine the high specificity of CRISPR-Cas systems with isothermal amplification techniques to provide rapid diagnostic tests at the point-of-care. Specifically, SHERLOCK combines isothermal amplification with Cas13a cleavage, where the guide RNA-Cas13a complex activates after specific binding to the target sequence. It then engages in collateral cleavage of nearby reporter RNA that is coupled to a quenched fluorophore, providing a signal that indicates pathogen detection (left side). In DETECTR, CRISPR guide RNA-Cas12a complexes activate after binding to target single-stranded DNA or dsDNA. Active Cas12a engages in indiscriminate cleavage of single-stranded DNA that is coupled to a fluorescent reporter or lateral flow assay (LFA) (right side).

In the Cas-based assay SHERLOCK, DNA or RNA is first amplified through isothermal techniques like RPA or reverse transcription RPA (RT-RPA), using a forward oligonucleotide that adds a T7 promoter to the target. Following this step, this promoter allows for RNA transcription of the amplicon, which is then recognized and bound by a complex of Cas13a and a crRNA that is complementary to the target sequence.^109^ The activation of Cas13a allows the cleavage both the target RNA by cis cleavage and, in a target-dependent manner, the ssRNA reporter molecules by *trans* cleavage. When cleaved, the ssRNA reporter allows the separation of the fluorophore from the quencher, resulting in an optical signal (e.g., fluorescence).^109^ In DETECTR, Cas12a is guided to dsDNA targets by a complementary crRNA, triggering collateral cleavage of short ssDNA reporters carrying a quencher and a fluorophore. Similar to SHERLOCK-based technology, target recognition, and reporter cleavage results in a fluorescence signal (Figure 5). These approaches have been reviewed in detail elsewhere.^109^ Given their versatility and easy adaptability, these CRISPR-based systems have been used to detect a range of RNA and DNA pathogens including SARS-CoV-2,^112^^,^^113^^,^^115^^,^^116^^,^^117^^,^^118^^,^^119^ Ebola virus,^120^ Zika virus,^99^^,^^110^ dengue virus,^110^ and Japanese encephalitis virus.^121^

In response to the current outbreak, CRISPR-based systems have been developed for MPXV diagnostics.^122^^,^^123^ In one of the first detection methods, Sui and co-workers developed a CRISPR system that was able to detect the MPXV DNA by using fluorescence readout.^122^ In this study, the authors found that the FAM fluorescent signal was detectable in 2 min and a strong signal was achieved within 10 min, indicating that the system has potential to apply in the field.^122^ In another similar report, Singh and co-workers designed a CRISPR-Cas12a-based system to detect MPXV, achieving a high sensitivity and specificity to detect synthetic DNA.^123^ Despite these promising findings, CRISPR/Cas-based diagnostic methods are not currently used in reference laboratories and need further implementation.

#### Serological methods

**Figure 6:**
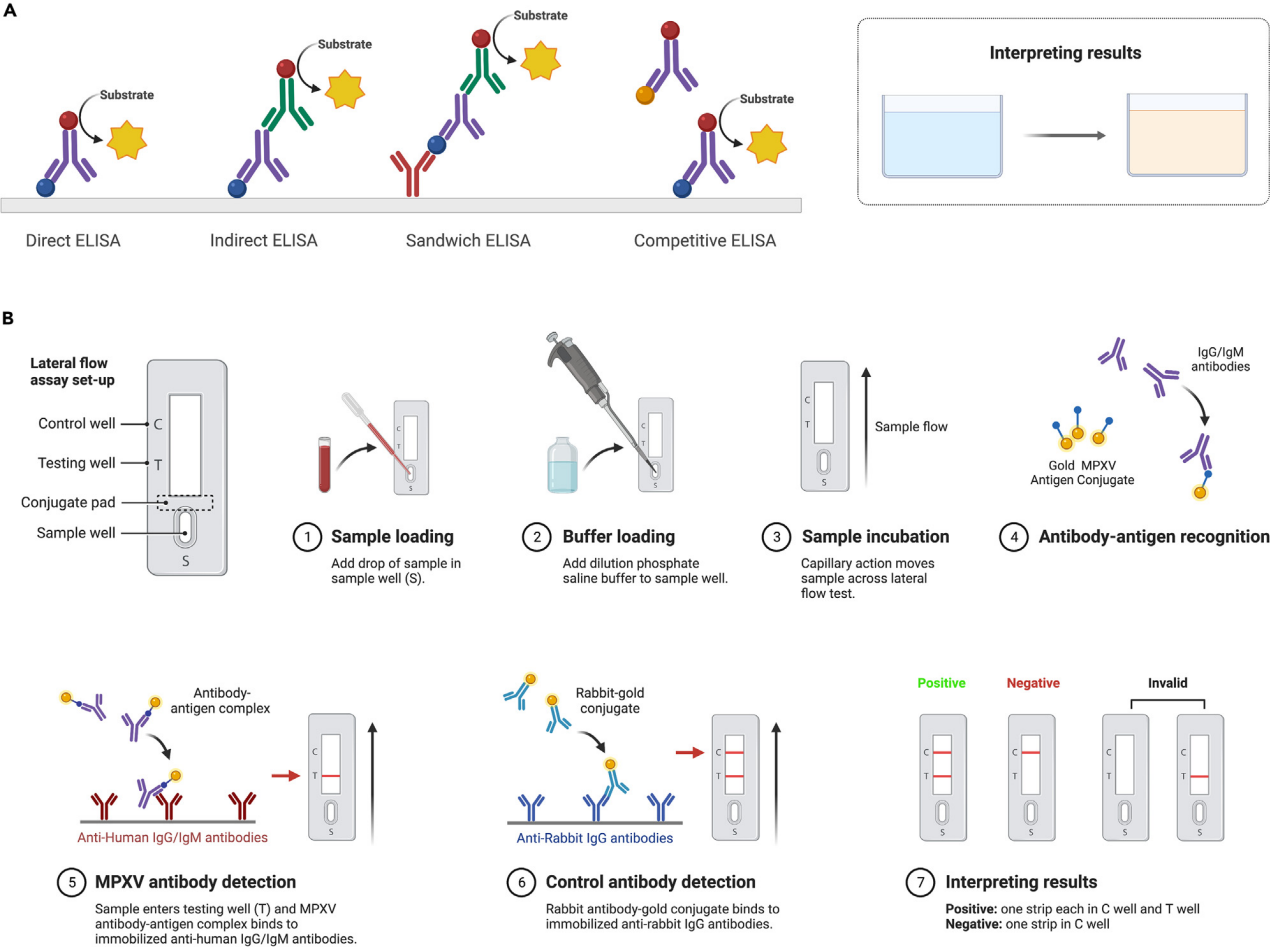
Immune-based assays to confirm mpox infection Different strategies (e.g., direct ELISA, indirect ELISA, sandwich ELISA, and competitive ELISA) to confirm mpox infection in suspected cases (A). Principle and common steps for antigen recognition and MPXV antibody detection using lateral flow assays (LFAs): (1), sample loading; (2), buffer loading; (3) sample incubation; (4) antibody-antigen recognition; (5), MPXV antibody detection; (6), control antibody detection; and (7), interpretation of results. Importantly, serological assays may show negative results for individuals who have been recently infected (B).

Serological assays have been developed to investigate the immunological response against mpox infection, with a focus on the detection of the related patient immunoglobulin M (IgM) and IgG antibodies (Figure 6). These serological tests include enzyme-linked immunosorbent assay (ELISA), lateral flow assays (LFAs), plaque reduction neutralization testing (PRNT), hemagglutination inhibition, complement fixation, immunofluorescence assay (IFA), and immunohistochemistry.^124^^,^^125^^,^^126^^,^^127^ Despite these utility of these techniques for seroprevalence and vaccine efficacy studies,^125^ they remain of limited value for determination and differentiation of orthopoxviruses species and diagnosis of mpox acute infection. In particular, cross-reactivity between orthopoxviruses represents one of the most critical limitations of serological-based methods for the diagnosis of mpox in clinical practice, especially in areas where there is a circulation of other orthopoxviruses or in individuals who are asymptomatic.^128^ Recent vaccination may also interfere with serological testing of suspected cases, for example, vaccination against smallpox can often provide some protection against mpox infection.^21^^,^^129^^,^^130^ It is for this reason that, in smallpox vaccinated individuals, the use of IgG as a diagnostic indicator can be a technical challenge due to the longevity of IgG responses and cross-reactivity with other orthopoxviruses.^131^ The detection of IgM antibodies from recent acute patients or related IgG antibodies from paired serum samples, collected at least 21 days apart, with the first being collected during the first week of the disease, can aid diagnosis if tested samples yield inconclusive findings.^21^ In summary, for these reasons, antibody detection from plasma or serum should not be used independently for mpox diagnosis.^21^

Considering the current arsenal of serological methods for the diagnosis of mpox, it is evident that many challenges still need to be overcome on the road to diagnostic tools that can provide reliable and accurate results. These challenges include the development of low-cost, high-capacity, and field-deployable serological diagnostics that are able to differentiate infection caused by different orthopoxviruses. To reduce cross-reactivity between orthopoxviruses, some studies have used methodological approaches, such as radioimmunoassays and neutralization assays.^132^ Despite promising results, these strategies are complex and would face several limitations in POC diagnostic settings. To meet this need, we envision the development of a diverse list of strategies that will elevate the next generation of serological methods for infectious disease testing. Such key features include: 1) minimum sample handling/processing; 2) less time-consuming; 3) low-cost involved; 4) easy-to-operate without expensive equipment, electricity, or extensive expertise; 4) enable high-capacity testing; 5) ability to be transported without a cold chain; 6) provide remote data access. When combined, these characteristics have the potential to promote de-centralization of diagnosis and, consequently, could be used for real-time monitoring and provide increased diagnostic capacity.

## Wastewater-based epidemiology of mpxv

Wastewater-based epidemiology (WBE) is a relatively new methodology based on chemical analysis of biomarkers and pollutants in raw wastewater and has the potential to provide qualitative and quantitative data about the exposure to hazards , such as pollutants within a particular community.^133^ Moreover, WBE provides an opportunity for near real-time, cost-effective monitoring of community-level transmission of specific pathogens and, consequently, allows for the estimate of disease burden in the community based on the biomarkers in wastewater.^134^ During the COVID-19 pandemic course, for example, several studies have used the WBE as a surveillance tool in order to identify hotspots of the disease through SARS-CoV-2 RNA detection.^135^^,^^136^ With the mpox outbreak ongoing, recent reports have documented the detection of the MPXV genome in sewersheds around the world, including in the USA,^137^ France,^138^ and Spain.^139^

## Concluding remarks and future directions

In the past two decades, our global society has experienced several public health emergencies caused by viral pathogens, including SARS-CoV, MERS-CoV, Ebola virus, Zika virus, and SARS-CoV-2. The spread of these viruses in the human population has motivated the development of rapid and accurate diagnostic testing that can be conducted in the field, especially in limited-resource settings. Real-time PCR is currently used as the reference molecular technique to diagnose these infections and current mpox patients. However, this lab-based method is relatively expensive, requires technical expertise, and utilizes an instrument that is incompatible with use in remote and low-resource areas, where surveillance and containment are critically needed. In less than three years, the COVID-19 pandemic has taught several lessons and brought rapid advancements in terms of diagnostic technologies for rapid, affordable, and accurate diagnose use at home or in the field. Certainly, these lessons from the COVID-19 pandemic will be crucial in confronting the present multi-country mpox outbreak and future public health biothreats.

Through this review, we have provided an overview of the rapidly expanding diagnostic technologies available to address our emerging need for agile and deployable diagnostics. In terms of patient-centric testing for mpox, there is a need for a low-cost diagnostic assay that is combined with simple sample-preparation workflows, robust detection output strategies, and remote data access.^109^

As a final thought, such newly developed diagnostic assays will need, of course, to be validated using patient samples in comparison side-by-side with the real-time PCR. After clinical implementation, diagnostic assays should be monitored over time to elucidate the performance of diagnostic tools under real-life settings. In addition, several analytical parameters should be assessed to evaluate the diagnostic performance of developed tests, including diagnostic sensitivity, diagnostic specificity, positive predictive value (PPV), negative predictive value (NPV), and overall accuracy.^140^
